# India Is Overtaking China as the World’s Largest Emitter of Anthropogenic Sulfur Dioxide

**DOI:** 10.1038/s41598-017-14639-8

**Published:** 2017-11-09

**Authors:** Can Li, Chris McLinden, Vitali Fioletov, Nickolay Krotkov, Simon Carn, Joanna Joiner, David Streets, Hao He, Xinrong Ren, Zhanqing Li, Russell R. Dickerson

**Affiliations:** 1 Earth System Science Interdisciplinary Center, University of Maryland, College Park, MD 20742 USA; 2 Atmospheric Chemistry and Dynamics Laboratory, NASA Goddard Space Flight Center, Greenbelt, MD 20771 USA; 3 Air Quality Research Division, Environment and Climate Change Canada, Toronto, M3H 5T4 Canada; 4 Department of Geological and Mining Engineering and Sciences, Michigan Technological University, Houghton, MI 49931 USA; 5 Energy Systems Division, Argonne National Laboratory, Argonne, IL 60439 USA; 6 Department of Atmospheric and Oceanic Science, University of Maryland, College Park, MD 20742 USA; 7 Air Resources Laboratory, National Oceanic and Atmospheric Administration, College Park, MD 20740 USA; 8 State Key Laboratory of Earth Surface Processes and Resource Ecology and College of Global Change and Earth System Science, Beijing Normal University, Beijing, 100875 China

## Abstract

Severe haze is a major public health concern in China and India. Both countries rely heavily on coal for energy, and sulfur dioxide (SO_2_) emitted from coal-fired power plants and industry is a major pollutant contributing to their air quality problems. Timely, accurate information on SO_2_ sources is a required input to air quality models for pollution prediction and mitigation. However, such information has been difficult to obtain for these two countries, as fast-paced changes in economy and environmental regulations have often led to unforeseen emission changes. Here we use satellite observations to show that China and India are on opposite trajectories for sulfurous pollution. Since 2007, emissions in China have declined by 75% while those in India have increased by 50%. With these changes, India is now surpassing China as the world’s largest emitter of anthropogenic SO_2_. This finding, not predicted by emission scenarios, suggests effective SO_2_ control in China and lack thereof in India. Despite this, haze remains severe in China, indicating the importance of reducing emissions of other pollutants. In India, ~33 million people now live in areas with substantial SO_2_ pollution. Continued growth in emissions will adversely affect more people and further exacerbate morbidity and mortality.

## Introduction

China and India are the top two consumers of coal in the world^1^. Coal typically contains a few percent of sulfur by weight, and its combustion emits large amounts of SO_2_, a toxic air pollutant. SO_2_ forms sulfate aerosols, the principal component of the historic “London Smog” and a major contributor to the two countries’ current haze problem^2,3^ that causes over one million premature deaths each year^4,5^. Sulfate commonly makes up >10% of the fine particles in China^2^ and India^3^, often much more during heavy pollution episodes^6^. To predict and mitigate air pollution, air quality models require accurate information on the emissions of SO_2_ and other pollutants. In the conventional approach, one compiles bottom-up emission inventories based on activity rates and emission factors. These inventories are normally updated every 3–5 years^7^ and often have to be projected for very recent years. Substantial uncertainties can therefore exist in the estimated or projected emissions, especially for regions experiencing rapid changes in economy and environmental regulations such as China^8^ and India^9^.

Advances in satellite measurements have yielded new data and techniques that help to evaluate and improve bottom-up inventories^10–13^. For SO_2_, the Ozone Monitoring Instrument (OMI) has been particularly useful owing to its superior ground resolution^14^. OMI SO_2_ measurements uncovered the first evidence that China had started to reduce emissions through the installation of flue gas desulfurization devices^15^, and also observed large changes in SO_2_ emissions from power plants in the U.S.^16,17^ and India^9^. More recently, a new technique that combines wind and improved SO_2_ data was employed to develop an OMI-based emission catalogue for nearly 500 sources around the globe^18–21^. This technique enabled the detection of ~40 sources missing from the conventional bottom-up inventories^18^ and provided the first emission estimates for a number of degassing volcanoes in remote locations^22^.

Here we analyze OMI SO_2_ data to study the changes in SO_2_ pollution in China and India from 2005 to 2016. We examine several recent emission projections to determine whether our observed changes were predicted in any emission scenarios. To investigate the underlying causes for the different trends between China and India, we compare emissions to coal consumption. Finally, we investigate the implications of these changes in SO_2_ pollution in terms of their health impacts.

## Results

### Changes in SO_2_ loading

**Figure 1:**
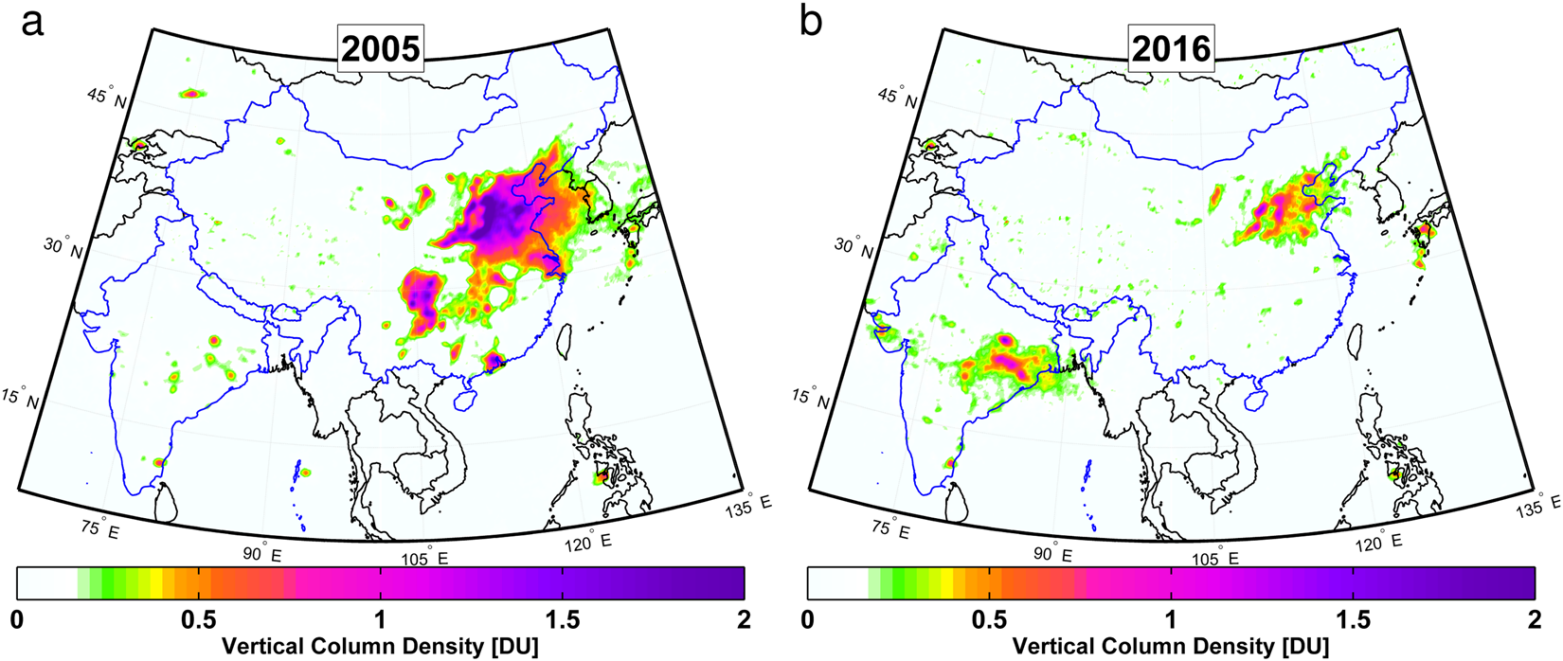
Changes in SO_2_ loading over India and China between 2005 and 2016. (**a**) Average SO_2_ vertical column amounts over India and China for 2005 from the OMI instrument on the Aura satellite, expressed in Dobson Units (1 DU = 2.69 × 10^16^ molecules cm^−2^). (**b**) Same as (**a**) but for 2016, showing significant increase and decrease of SO_2_ over India and China, respectively, during the 12-year span. The maps in the figure were generated by Chris McLinden using Matlab (version 2016a; https://www.mathworks.com/products/matlab.html).

For both China and India, OMI data show large differences in SO_2_ loading between 2005 and 2016, and in Fig. 1a, one can identify isolated hot spots with SO_2_ column amount >0.5 Dobson Units (DU, 1 DU = 2.69 × 10^16^ molecules cm^−2^) over India in 2005. Several are associated with large coal-fired power plants in the northeastern Indian states of Odisha, Jharkhand, and Chhattisgarh, the southeastern state of Tamil Nadu (which includes Chennai), and the western state of Maharashtra (which includes Mumbai). By 2016 (Fig. 1b), these hotspots in northeastern India have grown into a cluster covering a large area, likely due to emissions from new power plants constructed over the past decade^9,23^. SO_2_ columns in other parts of the country have also increased, particularly near Jamnagar on the west coast, where expansion of a large oil refinery and construction of the largest power plant in India took place in 2008–2012.

As for China, SO_2_ exceeded 0.5 DU over almost the entire North China Plain in 2005 (Fig. 1a). SO_2_ columns of >2 DU are found over Hebei and Henan, two provinces just east of the Taihang Mountains and home to numerous power plants as well as coking and cement industries. Additional SO_2_ hotspots can be seen over the populous Sichuan Basin, the Pearl River Delta in southern China (which includes Guangzhou and Hong Kong), the Yangtze River Delta in eastern China (which includes Shanghai), as well as Inner Mongolia. By 2016, almost no hotspots with SO_2_ >0.5 DU can be found outside of the North China Plain. Even for areas near the Taihang Mountains, the SO_2_ loading has generally decreased to below 1 DU (Fig. 1b).

### Changes in SO_2_ emissions

We estimate the annual SO_2_ emissions from China and India during 2005–2016 (Tables S1 and S2, Supplementary Material), by first summing up the sources in the OMI catalogue^21^ for the two countries. The catalogue includes emissions estimated based on OMI data (see Methods) for 47 sources in India and 82 sources in China. One caveat is that OMI can only detect large point sources and globally, the catalogue represents approximately 50% of all emissions in bottom-up inventories^18^. Here we compare the OMI-derived catalogue emissions with those from several recent regional inventories (Table 1). For China, the ratio between OMI catalogue emissions and inventories ranges within 40–62%; for India, this ratio is 36–48%.

**Table 1:** Recent bottom-up estimates and projections of SO_2_ emissions for China and India.

	Emission Estimates^*^ (Mt yr^−1^)							Emission Projections^**^ (Mt yr^−1^)				Source
	2005	2006	2007	2008	2009	2010	2011	2015	2020	2025	2030	
India						7.8 (48%)		10.4	12.4–12.9	2.9–15.3	3.3–18.8	^37^
	6.4 (39%)								16.0		31.5	^38^
	5.8 (43%)				7.5 (45%)	7.9 (47%)		9.1–9.7	9.3–11.9	8.6–13.5	8.7–15.6	^34^
	6.8 (36%)	7.2 (40%)	7.7 (41%)	8.4 (38%)	9.1 (38%)	9.5 (39%)	10.1 (36%)					^25^
				8.0 (40%)		8.8 (42%)						^39^
China	34.4 (51%)								33.3		32.9	^38^
	30.4 (58%)				33.2 (40%)	33.8 (45%)		33.6–34.6	28.8–33.3	22.4–30.2	17.7–27.7	^34^
	32.4 (54%)	33.3 (58%)	32.6 (62%)	31.2 (55%)	31.0 (43%)	29.8 (51%)	29.1 (60%)					^25^
				32.1 (53%)		30.8 (49%)						^39^
	28.6 (61%)								22.9–33.0			^40^
	28.7 (61%)					24.4 (62%)			15.7–29.1		8.3–30.7	^28^
						27.7 (55%)		19.6–33.8	19.6–36.3	16.6–37.8	15.5–38.1	^41^
	32.3 (54%)	33.2 (58%)	32.3 (62%)	31.3 (55%)								^24^
		31.0 (62%)										^42^

^*^Percentages in parentheses are the fraction of bottom-up emissions observed by OMI. The fraction for China is 40–62%, with a mean of 55%. The fraction for India is 36–48%, with a mean of 41%.

^**^Lower end of the ranges typically represents emission scenarios with stricter emission control and energy policies that facilitate the shift to alternate energy sources rather than coal. Higher end typically represents emission scenarios with current environmental and energy policies (business as usual).

**Figure 2:**
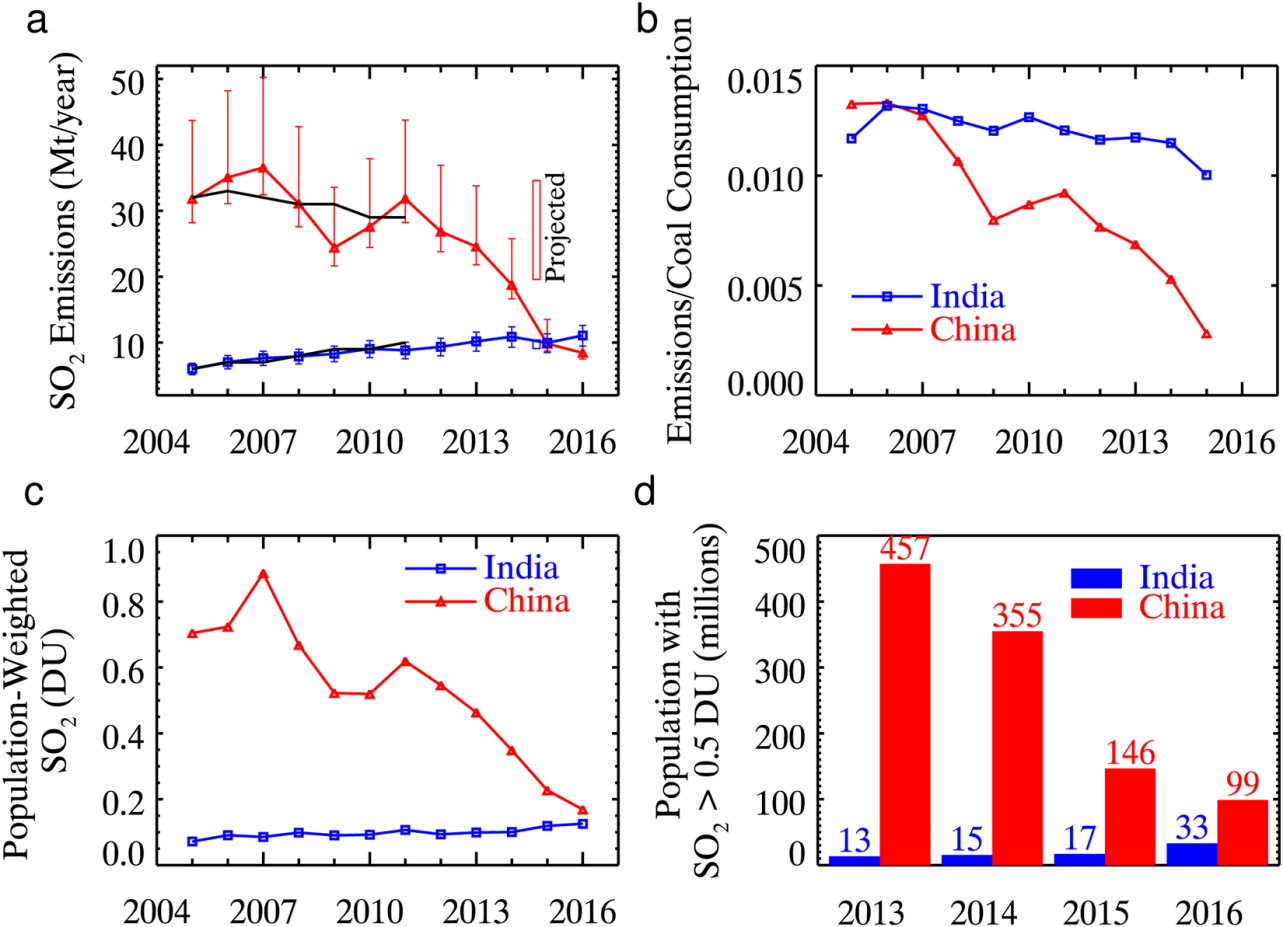
Emissions, loading, and potential impact of SO_2_ in India and China. (**a**) Total annual SO_2_ emissions for India and China during 2005–2016 based on OMI measurements. To account for the sources that are undetectable by OMI, the top-down estimates from the OMI emission catalogue are normalized using the average ratio (55% for China, 41% for India) between the catalogue and various bottom-up inventories in Table 1. The lower and upper bounds of the error bars are the same OMI estimates normalized with the maximum and minimum ratios between OMI and bottom-up inventories, respectively. Black lines represent annual emissions from one of the bottom-up inventories^25^. Vertical bars show the range of projected emissions for 2015 (Table 1). (**b)** The ratio of the unnormalized OMI emission estimates to coal consumption during 2005–2015 (unit: tonne SO_2_/tonne oil equivalent). (**c**) Population-weighted SO_2_ loading in Dobson Units. (**d**) Population living in areas with annual mean SO_2_ of at least 0.5 DU during 2013–2016.

We then use the average of these ratios to adjust or normalize the OMI-derived emissions to reflect the national total. As shown in Fig. 2a, the normalized estimates reveal opposite trends in China and India. The emissions from China peaked at 36.6 Mt (10^6^ tonnes) yr^−1^ in 2007 and have since been on a generally decreasing trajectory. At 8.4 Mt yr^−1^, the level in 2016 is 26% of that in 2005 (31.8 Mt yr^−1^). The decrease reflects stricter pollution control measures, coupled with a gradual shift to other, non-coal-based energy sources, and the recent slowdown of the Chinese economy. Since the early 2000s, the Chinese government has introduced, for example, policies to reduce SO_2_ emissions^24^ and a new national air quality standard for fine particles^8^. Electricity generation in China grew by more than 100% during 2005–2015, but coal consumption increased by ~50%^1^. The brief period of emission growth in 2009–2011 can probably be attributed to government stimulus in response to the global financial crisis of 2007–2008.

The estimated emissions for India, on the other hand, indicate relatively steady growth throughout the entire period. For 2016, the emissions from India (11.1 Mt yr^−1^, 9.5–12.6 Mt yr^−1^ considering the range of OMI/bottom-up ratios) are at approximately the same level as China (7.5–11.6 Mt yr^−1^). If the current trends continue, India will emit significantly more SO_2_ than China in the coming years. For both countries, the ratio between the OMI catalogue emissions and total emissions may change over time. We also estimate SO_2_ emissions based on the OMI-observed total SO_2_ mass, calculated from observations over the entire country and therefore less affected by the detection limit. We arrive at the same conclusion that India is becoming, if it is not already, the world’s top SO_2_ emitting country (Tables S3 and S4).

It is enlightening to compare the OMI catalogue emissions with coal consumption (Fig. 2b). The ratio between the two is similar for China and India for 2005–2007 at ~0.012–0.013 tonne SO_2_/tonne oil equivalent. Considering that OMI observes ~50% of all SO_2_ sources, that ~70–90% of SO_2_ emissions are from coal^25^, and upon accounting for standard conversions (1 tonne oil equivalent = 1.4 tonnes coal), we arrive at an effective emission factor of 12–16 g SO_2_/kg coal. This is largely consistent with previously used values in bottom-up inventories^24,26^ and suggests little control on SO_2_ in either country before 2007. Since then, the ratio has remained virtually unchanged for India, implying continued absence of SO_2_ emission control^9^. The ratio for China, however, has dropped to ~0.002–0.003 (corresponding emission factor: 2–3 g SO_2_/kg coal), suggesting effective control measures that have eliminated ~80% of potential SO_2_ emissions.

Assuming that carbon makes up ~60–80% of coal by weight, our estimated emission factor for 2015 implies a molar ratio of ~5–9 × 10^−4^ between SO_2_ and CO_2_ emitted from coal combustion in China. This is comparable with the recently measured ΔSO_2_/ΔCO_2_ ratio of ~3–10 × 10^−4^ (ΔSO_2_ and ΔCO_2_ represent the observed enhancements within plumes over background levels) in the boundary layer over Hebei in spring 2016 (Fig. S1), confirming the efficient SO_2_ removal in China. Satellite observations^27^ also point to a ~25% increase in NH_3_ over China during our study period. This relatively modest growth (as compared with the decrease in SO_2_) is partially attributable to reductions in SO_2_ as a sink for NH_3_. It also suggests that there must be excess NH_3_ and other alkaline cations that neutralize sulfate; otherwise the growth rate in NH_3_ would have been much greater. Indeed recent measurements in northern China^6^ seem to indicate complete neutralization of sulfate and nitrate in aerosols.

In Table 1, we examine projections of SO_2_ emissions from several studies published between 2009 and 2015. For India, the projected emissions for 2015 are 9.1–10.4 Mt yr^−1^, close to our estimate of 8.5–11.3 Mt yr^−1^ (Table S1). For China, the projected emissions for 2015 (19.6–33.8 Mt yr^−1^) are a factor of 1.5–4 greater than our estimate (8.7–13.5 Mt yr^−1^). In fact, all but one study predicted that SO_2_ emissions from China would still exceed 15 Mt yr^−1^ even in 2030. In the only exception^28^ (8 Mt yr^−1^ in 2030), it is assumed that lifestyle-changing energy policies and the most efficient emission control technology would be fully implemented in China. The difference between our observation and projections suggests that there are currently much more efficient SO_2_ controls in China than assumed in the various emission scenarios.

### Population exposure to SO_2_ pollution

Population-weighted SO_2_ loading (Fig. 2c, Table S5) closely follows OMI-estimated emissions. Over the past 10 years, the SO_2_ loading over China decreased by a factor of five, from 0.89 DU in 2007 to 0.17 DU in 2016. At the same time, the loading over India climbed by nearly 50%, reaching 0.13 DU in 2016. There is no simple relationship between the OMI-observed column amount and the concentration at ground level. If we assume that all the SO_2_ is within the lowest 1000 m of the atmosphere and well mixed at 1:30 pm local time (OMI overpass time), an SO_2_ column of 0.5 DU corresponds to a mass concentration of ~14 μg m^−3^. Given that the World Health Organization’s guideline for SO_2_ is 20 μg m^−3^ (for a 24-hour mean), column amounts of 0.5–1 DU represent sufficiently high SO_2_ loading to adversely affect human health both as a toxic gas and as a precursor to sulfate aerosols. In China, over 450 million people were exposed to >0.5 DU of SO_2_ in 2013, but this number decreased to 99 million in 2016 (Fig. 2d). Similarly, the population exposed to >1.0 DU of SO_2_ decreased from ~190 million in 2013 to 13 million in 2016, a remarkable drop of over 90% (Table S6). As for India, 13 (0.7) million people were exposed to >0.5 (1.0) DU of SO_2_ in 2013. In just three years, this has grown to 33 (3.8) million people (Table S7).

## Discussion

Our findings have important implications for future environmental policies in both countries. Despite the large reductions in SO_2_, haze in China remains a severe environmental issue^29^. This may be partly due to the shift in the thermodynamic equilibrium of the sulfate-nitrate-ammonium system^6^. It will be critical to better understand the benefits of SO_2_ reductions before viable and balanced policies can be devised to further improve air quality in China. To a certain extent, the impact of SO_2_ emissions is presently limited in India, as SO_2_ loading is relatively low over the densely populated Indo-Gangetic Plain. But this may change as the demand for electricity continues to grow. In the various Representative Concentration Pathways for the latest Assessment Report (AR5) by the Intergovernmental Panel on Climate Change^30^, SO_2_ emissions from Asia were projected to increase until the 2020s before starting to decrease. The sooner-than-expected reductions in SO_2_ could also accelerate regional warming, as they would reduce the loading of sulfate aerosols that scatter sunlight and partially offset the warming effects of greenhouse gases.

## Methods

### OMI SO_2_ data

SO_2_ data used in this study were retrieved from earthshine radiances in the wavelength range of 310.5–340 nm measured by the Ozone Monitoring Instrument^31^ (OMI) aboard the NASA Aura spacecraft. The results are in Dobson Units (1 DU = 2.69 × 10^16^ molecules cm^−2^), and represent the estimated total number of SO_2_ molecules in the entire atmospheric column above a unit area (or simply, column amount). The current retrieval algorithm applies a principal component analysis technique to OMI radiances to minimize spectral interferences and maximize the quality of SO_2_ data. A detailed description of the retrieval technique can be found elsewhere^20,32^. Because the OMI SO_2_ sensitivity varies with altitude, the retrieved total column amount depends on the assumed vertical distribution of SO_2_. Several different fixed SO_2_ profiles are assumed in operational OMI retrievals. The present study uses version 1.3 level 2 (orbital level) OMI retrievals assuming that all SO_2_ is in the planetary boundary layer (PBL, or the lowest 1 km of the atmosphere).

For the present study, OMI pixels with a radiative cloud fraction >0.2 or solar zenith angle >70**°** were excluded from data analysis. Data from OMI cross-track positions (or rows) affected by the row anomaly (http://projects.knmi.nl/omi/research/product/rowanomaly-background.php) or near the edge of the swath (rows 1–10 and 51–60) were also excluded. Additionally, data from days potentially influenced by large transient volcanic plumes were excluded. Details on the data filtering can be found elsewhere^18^. The SO_2_ columns from the remaining OMI pixels were then averaged to a spatial resolution of 0.1** × **0.1**°** for the maps in Fig. 1.

### OMI-based SO_2_ source detection and estimate

The methods for source detection and emission estimate are based on a previously described algorithm^18,19,21^ that combines satellite measurements with reanalysis wind data (ECMWF interim reanalysis^33^ was used here). Wind information is matched with each OMI pixel. Emissions from about 500 continuously emitting point sources (or clusters of sources in close proximity), including 47 in India and 82 in China, are derived from OMI and wind data by tracking the downwind decay of the plumes. These sources have estimated SO_2_ emissions ranging from about 30 kt yr^−1^ to more than 4000 kt yr^−1^. Due to the coarse spatial resolution of OMI (relative to a point source) and the limited precision of individual SO_2_ column observations, data spanning a year are analyzed together using a wind rotation scheme to align the wind vectors of all overpasses considered^19^. The emissions were estimated by fitting OMI columns to a plume function^19^ consisting of coordinates, wind speed and direction, with a single parameter representing the total mass^18,21^. Other fitting parameters, including an effective lifetime (5.9 hours), are specified^21^. Emissions are then calculated as the ratio of mass to lifetime, effectively assuming a steady-state. The operational OMI retrievals use an effective air mass factor of 0.36 for all locations. In the emission estimate algorithm, OMI data for each emission source were adjusted using an air mass factor calculated for the location based on its elevation, surface albedo and sun/viewing geometry to better represent OMI sensitivities to the local source^18,21^.

Wet removal of pollutants by the summer monsoon rainfall causes a strong seasonality in air pollution in India, especially for aerosols^35^. OMI generally also observes smaller SO_2_ columns over India during summer months (see monthly maps at https://avdc.gsfc.nasa.gov/pub/data/satellite/Aura/OMI/V03/L3/OMSO2m/Monthly_mean_jpeg/). This seasonality may be partially attributed to the washout effects and the shorter lifetime of SO_2_ in summer, but may also reflect reduced coverage by OMI due to increased cloud cover during the monsoon. For OMI-based emission estimates, since pixels with a small cloud fraction from an entire year are analyzed, the collection of data used to derive emissions for a given source may be more representative of non-summer conditions. The impact of this seasonal change in sampling on estimated emissions is currently unclear, but it is unlikely to significantly affect their long-term trend.

### Aircraft measurements of ΔSO_2_/ΔCO_2_ ratio

Between 8 May and 11 June 2016, a twin engine Y-12 research aircraft was flown on 11 missions over the heavily industrialized Hebei Province of China. A modified, commercial pulsed-fluorescence detector (TEI Model 43 C) was used to measure ambient SO_2_. A Picarro cavity ring-down spectrometer (Model G2401-m) was used to measure CO_2_. Profiles were flown from near the surface to the top of the planetary boundary layer, at ~1500 m above ground. The ΔSO_2_/ΔCO_2_ ratio was determined from the deviation from background in plumes. Only data with significant correlation between ∆SO_2_ and ∆CO_2_ (R^2^ > 0.6) are included in Fig. S1.

### Population data and population exposure to SO_2_ pollution

Population data for 2005, 2010, and 2015 from the Gridded Population of the World, Version 4 (GPWv4)^36^ were used in this study. For each of the three years, the (30 arc seconds) GPWv4 population count and nation identifier data were used to calculate the counts of Chinese and Indian population for each grid cell in Fig. 1. An annual growth rate was then estimated for each grid cell between 2005 and 2010, and between 2010 and 2015, to interpolate population data to other years.

With OMI SO_2_ (*Ω*) and population count (*P*) data now on the same grid, the population-weighted SO_2_ column amount (*Ω*_*w*_) for the entire domain with *n* grid cells can be calculated as:

1 $${{\rm{\Omega }}}_{w}=\frac{\sum _{i=1}^{i=n}{P}_{i}{{\rm{\Omega }}}_{i}}{\sum _{i=1}^{i=n}{P}_{i}},$$

where *P*_*i*_ and *Ω*_*i*_ are population count and OMI SO_2_ column for the *i*^*th*^ grid cell, respectively.

### Data Availability

Level 2 Principal Component Analysis SO_2_ data from OMI are available from the Goddard Earth Science Data and Information Service Center (http://disc.sci.gsfc.nasa.gov/). Wind reanalysis data are available from ECMWF (http://apps.ecmwf.int/datasets/data/interim-full-daily). Derived SO_2_ emissions are available from the global SO_2_ monitoring website at NASA Goddard Space Flight Center (https://so2.gsfc.nasa.gov). The GPWv4 population data are available from the Socioeconomic Data and Applications Center (SEDAC) in NASA’s Earth Observing System Data and Information System and hosted by Center for International Earth Science Information Network at Columbia University (http://sedac.ciesin.columbia.edu/data/collection/gpw-v4). Aircraft measurements acquired during the ARIAS campaign are available upon request from X. Ren (ren@umd.edu).

## Electronic supplementary material

Supplementary Information
