# Author Correction: India Is Overtaking China as the World’s Largest Emitter of Anthropogenic Sulfur Dioxide

**DOI:** 10.1038/s41598-018-26657-1

**Published:** 2018-06-06

**Authors:** Can Li, Chris McLinden, Vitali Fioletov, Nickolay Krotkov, Simon Carn, Joanna Joiner, David Streets, Hao He, Xinrong Ren, Zhanqing Li, Russell R. Dickerson

**Affiliations:** 1 Earth System Science Interdisciplinary Center, University of Maryland, College Park, MD 20742 USA; 2 Atmospheric Chemistry and Dynamics Laboratory, NASA Goddard Space Flight Center, Greenbelt, MD 20771 USA; 3 Air Quality Research Division, Environment and Climate Change Canada, Toronto, M3H 5T4 Canada; 4 Department of Geological and Mining Engineering and Sciences, Michigan Technological University, Houghton, MI 49931 USA; 5 Energy Systems Division, Argonne National Laboratory, Argonne, IL 60439 USA; 6 Department of Atmospheric and Oceanic Science, University of Maryland, College Park, MD 20742 USA; 7 Air Resources Laboratory, National Oceanic and Atmospheric Administration, College Park, MD 20740 USA; 8 State Key Laboratory of Earth Surface Processes and Resource Ecology and College of Global Change and Earth System Science, Beijing Normal University, Beijing, 100875 China

Correction to: *Scientific Reports* 10.1038/s41598-017-14639-8, published online 09 November 2017

This Article contains multiple errors.

In the original version of this Article, an affiliation for Zhanqing Li was omitted. The correct affiliations for Zhanqing Li are given below:

Earth System Science Interdisciplinary Center, University of Maryland, College Park, MD, 20742, USA

Department of Atmospheric and Oceanic Science, University of Maryland, College Park, MD, 20742, USA

State Key Laboratory of Earth Surface Processes and Resource Ecology and College of Global Change and Earth System Science, Beijing Normal University, Beijing, 100875, China

Furthermore, the Acknowledgements section in this Article was incomplete.

“We thank the NASA Earth Science Division (ESD) Aura Science Team program for funding of OMI SO2 product development and analysis (Grant # 80NSSC17K0240). The Dutch and Finnish built OMI instrument is part of the NASA’s Earth Observing System (EOS) Aura satellite payload. The OMI project is managed by the Royal Meteorological Institute of the Netherlands (KNMI) and the Netherlands Space Agency (NSO). We thank Dr. Steve Fetter (University of Maryland) for helpful comments. We thank NSF for supporting aircraft measurements over China presented in this study (Grant # 1558259). C.L. acknowledges partial support from NASA’s Earth Science New Investigator Program (Grant # NNX14AI02G).”

now reads:

“We thank the NASA Earth Science Division (ESD) Aura Science Team program for funding of OMI SO2 product development and analysis (Grant # 80NSSC17K0240). The Dutch and Finnish built OMI instrument is part of the NASA’s Earth Observing System (EOS) Aura satellite payload. The OMI project is managed by the Royal Meteorological Institute of the Netherlands (KNMI) and the Netherlands Space Agency (NSO). We thank Dr. Steve Fetter (University of Maryland) for helpful comments. We thank the National Science Foundations of China (91544217) and US (Grant # 1558259) for supporting aircraft measurements over China presented in this study. C.L. acknowledges partial support from NASA’s Earth Science New Investigator Program (Grant # NNX14AI02G).”

These errors have now been corrected in the PDF and HTML versions of the Article and in the accompanying Supplementary Information file.

