# Supplementary material for: India Is Overtaking China as the World’s Largest Emitter of Anthropogenic Sulfur Dioxide
Source: Sci Rep. 2017 Nov 9;7:14304. doi: 10.1038/s41598-017-14639-8 (PMC5680191; doi:10.1038/s41598-017-14639-8)
Supplement: Supplementary file 1 — Supplementary Information [file 41598_2017_14639_MOESM1_ESM.pdf]

Supplementary Material

India Is Overtaking China as the World's Largest Emitter of Anthropogenic Sulfur Dioxide

Can Li[1,2], Chris McLinden[3], Vitali Fioletov[3], Nickolay Krotkov[2], Simon Carn[4], Joanna Joiner[2], David Streets[5], Hao He[6], Xinrong Ren[6,7], Zhanqing Li[1,6,8], Russell R. Dickerson[1,6]
[1]Earth System Science Interdisciplinary Center, University of Maryland, College Park, MD 20742, USA.
[2]Atmospheric Chemistry and Dynamics Laboratory, NASA Goddard Space Flight Center, Greenbelt, MD 20771, USA.
[3]Air Quality Research Division, Environment and Climate Change Canada, Toronto M3H 5T4, Canada.
[4]Department of Geological and Mining Engineering and Sciences, Michigan Technological University, Houghton, MI 49931, USA
[5]Energy Systems Division, Argonne National Laboratory, Argonne, IL 60439, USA.
[6]Department of Atmospheric and Oceanic Science, University of Maryland, College Park, MD 20742, USA.
[7]Air Resources Laboratory, National Oceanic and Atmospheric Administration, College Park, MD 20740, USA.
[8]State Key Laboratory of Earth Surface Processes and Resource Ecology and College of Global Change and Earth System Science, Beijing Normal University, Beijing, 100875, China.

Table S1. Total annual SO_2 emissions for China estimated based on the top-down OMI catalogue.

Year	2005	2006	2007	2008	2009	2010	2011	2012	2013	2014	2015	2016
OMI catalogue based (Mt)	17.5	19.3	20.1	17.1	13.4	15.2	17.5	14.8	13.5	10.3	5.4	4.6
OMI catalogue based, fraction corrected (Mt)*	31.8	35.1	36.6	31.1	24.4	27.6	31.8	26.8	24.6	18.8	9.8	8.4
OMI catalogue based, fraction corrected, min (Mt)**	28.2	31.1	32.4	27.6	21.7	24.4	28.2	23.8	21.8	16.6	8.7	7.5
OMI catalogue based, fraction corrected, max (Mt)***	43.7	48.2	50.3	42.8	33.6	37.9	43.8	36.9	33.8	25.8	13.5	11.6
Coal consumption (Mtoe)	1318	1448	1577	1603	1680	1743	1899	1923	1964	1949	1920	

*Correction based on the mean ratio (0.55) between the total OMI catalogue emissions for China and various bottom-up emission inventories in Table 1.
**Correction based on the maximum ratio (0.62) between the total OMI catalogue emissions for China and bottom-up emission inventories in Table 1.
***Correction based on the minimum ratio (0.40) between the total OMI catalogue emissions for China and bottom-up emission inventories in Table 1.

Table S2. Total annual SO_2 emissions for India estimated based on the top-down OMI catalogue.

Year	2005	2006	2007	2008	2009	2010	2011	2012	2013	2014	2015	2016
OMI catalogue based (Mt)	2.5	2.9	3.1	3.2	3.4	3.7	3.6	3.8	4.2	4.5	4.1	4.5
OMI catalogue based, fraction corrected (Mt)*	6.0	7.1	7.6	7.9	8.3	9.0	8.8	9.4	10.2	10.9	10.0	11.1
OMI catalogue based, fraction corrected, min (Mt)**	5.1	6.0	6.5	6.8	7.1	7.7	7.5	8.0	8.7	9.3	8.5	9.5
OMI catalogue based, fraction corrected, max (Mt)***	6.9	8.0	8.7	9.0	9.5	10.3	10.1	10.7	11.6	12.4	11.3	12.6
Coal consumption (Mtoe)	211	219	240	259	283	293	300	330	356	389	407	

*Correction based on the mean ratio (0.41) between the total OMI catalogue emissions for India and various bottom-up emission inventories in Table 1.
**Correction based on the maximum ratio (0.48) between the total OMI catalogue emissions for India and bottom-up emission inventories in Table 1.
***Correction based on the minimum ratio (0.36) between the total OMI catalogue emissions for India and bottom-up emission inventories in Table 1.

Table S3. Total annual SO_2 emissions for China estimated based on the total SO_2 mass observed by OMI and an assumed effective (e-folding) lifetime of 6 hours.

Year	2005	2006	2007	2008	2009	2010	2011	2012	2013	2014	2015	2016
OMI mass based (Mt)	29.1	32.9	37.3	31.2	20.5	22.2	29.1	22.2	21.1	15.8	9.8	8.5
OMI mass based, fraction corrected (Mt)*	32.0	36.1	41.0	34.3	22.5	24.3	32.0	24.3	23.2	17.3	10.7	9.4
OMI mass based, fraction corrected, min (Mt)**	25.3	28.6	32.4	27.1	17.8	19.3	25.3	19.3	18.3	13.7	8.5	7.4
OMI mass based, fraction corrected, max (Mt)***	47.0	53.0	60.1	50.3	33.1	35.7	47.0	35.7	34.0	25.4	15.8	13.8

*Correction based on the mean ratio (0.91) between the OMI mass-based emissions for China and various bottom-up emission inventories in Table 1.
**Correction based on the maximum ratio (1.15) between the OMI mass-based emissions for China and bottom-up emission inventories in Table 1.
***Correction based on the minimum ratio (0.62) between the OMI mass-based emissions for China and bottom-up emission inventories in Table 1.

Table S4. Total annual SO_2 emissions for India estimated based on the total SO_2 mass observed by OMI and an assumed effective (e-folding) lifetime of 6 hours.

Year	2005	2006	2007	2008	2009	2010	2011	2012	2013	2014	2015	2016
OMI mass based (Mt)	2.9	3.9	3.5	3.8	3.9	3.8	5.0	4.4	4.2	4.5	5.5	6.6
OMI mass based, fraction corrected (Mt)*	6.1	8.2	7.5	8.0	8.4	8.0	10.5	9.4	8.9	9.6	11.8	14.1
OMI mass based, fraction corrected, min (Mt)**	5.3	7.2	6.6	7.0	7.3	7.0	9.2	8.2	7.8	8.4	10.3	12.3
OMI mass based, fraction corrected, max (Mt)***	7.2	9.7	8.9	9.5	9.8	9.5	12.4	11.1	10.5	11.3	13.9	16.6

*Correction based on the mean ratio (0.47) between the OMI mass-based emissions for India and various bottom-up emission inventories listed in Table 1.
**Correction based on the maximum ratio (0.54) between the OMI mass-based emissions for India and bottom-up emission inventories in Table 1.
***Correction based on the minimum ratio (0.40) between the OMI mass-based emissions for India and bottom-up emission inventories in Table 1.

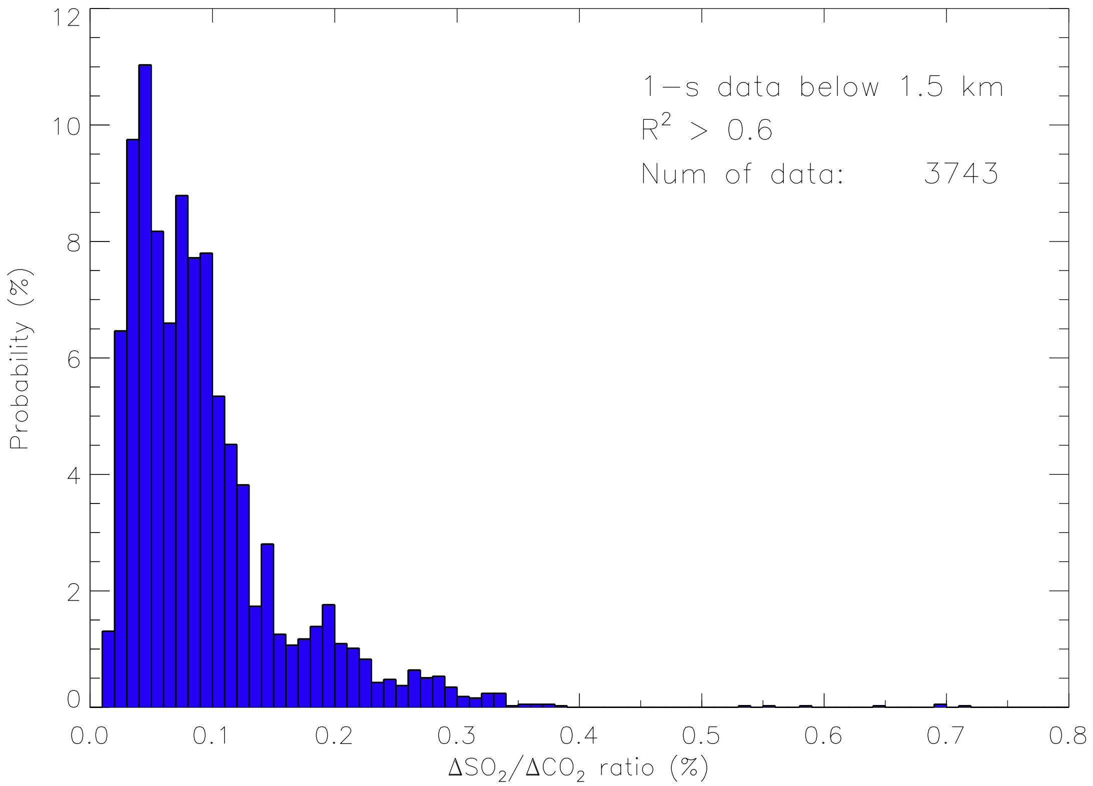

Figure S1. Histogram of $\Delta SO_2/\Delta CO_2$ ratios observed for plumes that were encountered below 1.5 km over Hebei, China during the ARIAS aircraft campaign in May 2016.

Table S5. Population-weighted mean SO_2 column amount in China and India (Unit: Dobson Unit).

Year	2005	2006	2007	2008	2009	2010	2011	2012	2013	2014	2015	2016
China	0.70	0.72	0.89	0.67	0.52	0.52	0.62	0.55	0.46	0.35	0.23	0.17
India	0.07	0.09	0.09	0.10	0.09	0.09	0.11	0.09	0.10	0.10	0.12	0.13

Table S6. Population exposed to different levels of annual SO_2 column amount in China (Unit: Million).

Year	2005	2006	2007	2008	2009	2010	2011	2012	2013	2014	2015	2016
0.5 DU	681.8	702.2	774.8	685.4	565.5	572.3	639.1	564.3	457.2	355.1	146.7	99.1
1.0 DU	373.6	371.9	524.6	365.6	213.7	197.3	277.8	250.9	190.0	88.0	13.6	13.0
1.5 DU	146.2	179.0	284.3	128.7	70.2	73.5	135.9	112.4	51.2	12.5	2.4	1.4
2.0 DU	54.1	54.8	112.3	42.3	22.9	18.3	44.6	34.1	19.6	2.9	0.0	0.0

Table S7. Population exposed to different levels of annual SO_2 column amount in India (Unit: Million).

Year	2005	2006	2007	2008	2009	2010	2011	2012	2013	2014	2015	2016
0.5 DU	3.8	4.4	5.2	7.0	6.4	8.9	9.4	10.6	13.4	15.4	17.0	33.3
1.0 DU	0.0	0.0	0.2	0.4	0.0	0.2	0.3	0.0	0.7	1.8	2.8	3.8
1.5 DU	0.0	0.0	0.0	0.0	0.0	0.0	0.0	0.0	0.0	0.0	0.2	0.2
2.0 DU	0.0	0.0	0.0	0.0	0.0	0.0	0.0	0.0	0.0	0.0	0.0	0.0
